# Effect of Integrated Lecture‐, Case‐, and Problem‐Based Learning on Critical Thinking and Clinical Competence in Gastrointestinal Surgery Trainees: A Randomized Controlled Trial

**DOI:** 10.1155/grp/8457575

**Published:** 2026-02-10

**Authors:** Jin Tang, Jing Yang, Junsong Yang, Shoujiang Wei

**Affiliations:** ^1^ Department of Gastrointestinal Surgery, Affiliated Hospital of North Sichuan Medical College, Nanchong, Sichuan Province, China, hospital-nsmc.com.cn; ^2^ Department of Rheumatology and Immunology, Beijing Anzhen Hospital, Capital Medical University, Nanchong Hospital-Nanchong Central Hospital, Nanchong, Sichuan Province, China, ccmu.edu.cn

**Keywords:** case-based learning, critical thinking, gastrointestinal surgery, medical education, problem-based learning

## Abstract

**Background:**

Enhancing clinical thinking and competence is central to gastrointestinal surgical training. This study is aimed at evaluating the effectiveness of an integrated instructional model combining lecture‐based learning (LBL), problem‐based learning (PBL), and case‐based learning (CBL) in gastrointestinal surgical training.

**Methods:**

This prospective randomized controlled trial was conducted at the Affiliated Hospital of North Sichuan Medical College, China, from September to November 2024. A total of 120 gastrointestinal surgical trainees, including final‐year medical interns and first‐year surgical residents, were randomly assigned (1:1:1) to the LBL, PBL + CBL, or integrated LBL + PBL + CBL groups using a computer‐generated sequence, with allocation concealed by an independent assistant. After completing an 8‐week standardized curriculum delivered by the same teaching team, the following outcomes were assessed: critical thinking ability using the Chinese version of the California critical thinking disposition inventory (CTDI‐CV); theoretical knowledge through written and oral examinations; learning efficiency (study time and task completion rate) and satisfaction; and clinical competence assessed via the miniclinical evaluation exercise (Mini‐CEX). Data were analyzed using IBM SPSS Statistics with one‐way analysis of variance, and statistical significance was set at *p* < 0.05.

**Results:**

The integrated LBL + PBL + CBL groups achieved the highest CTDI‐CV total scores, theoretical knowledge performance, and Mini‐CEX scores compared with the other groups (*p* < 0.05). Despite reporting the shortest weekly study time, the integrated group attained the highest task completion rate and learner satisfaction score (*p* < 0.05).

**Conclusion:**

The integrated LBL + PBL + CBL approach enhances efficiency and is well‐suited for advancing clinical competence in surgical training.

**Trial registration:**

Chinese Clinical Trial Registry (ChiCTR2000041569).

## 1. Introduction

The development of critical thinking and clinical decision‐making ability is a central goal of modern medical education, particularly in surgical disciplines where diagnostic complexity, intraoperative uncertainty, and high‐risk decisions are routine [1, 2]. In gastrointestinal surgery, where trainees are required to rapidly assess patient presentations, interpret complex imaging and laboratory results, and implement timely interventions, the demand for cognitive agility and integrative reasoning is especially high [3, 4]. Accordingly, there is an urgent need for instructional strategies that not only transmit foundational knowledge but also cultivate higher‐order thinking skills and practical clinical competence.

Traditionally, lecture‐based learning (LBL) has served as the cornerstone of medical and surgical education [5]. Its strengths lie in the efficient delivery of standardized content and the clear organization of knowledge domains. However, LBL is inherently passive, and multiple studies have suggested that it may not be effective in promoting long‐term retention, transfer of learning, or learner engagement [6]. Students often memorize information without truly understanding how to apply it in complex clinical settings, particularly in the context of rapidly evolving surgical scenarios [7].

To overcome the limitations of traditional didactic methods, educational theorists and curriculum designers have increasingly turned to active learning models, such as problem‐based learning (PBL) and case‐based learning (CBL). PBL, rooted in constructivist learning theory, emphasizes student autonomy, self‐directed learning, and collaborative problem‐solving [8, 9]. It encourages learners to formulate their own questions, identify relevant resources, and synthesize information to address authentic clinical challenges [9, 10]. CBL, although similarly learner‐centered, grounds instruction in real or simulated clinical cases [11]. It provides opportunities for students to integrate theoretical knowledge with diagnostic reasoning, decision‐making, and patient‐centered care [12]. Both approaches have demonstrated positive effects on students′ motivation, communication skills, and ability to think critically [13–15]. However, when implemented in isolation, PBL may lack content structure and comprehensive coverage, whereas CBL may not sufficiently encourage deep exploration of underlying pathophysiology or evidence‐based practice [16, 17].

In response, there has been growing interest in hybrid or integrated instructional models that combine LBL, PBL, and CBL [18, 19]. Theoretically, such integration allows educators to scaffold knowledge acquisition while promoting deep learning and clinical application. Learners first build a foundational understanding through lectures, then explore diagnostic frameworks and hypotheses through problem‐solving, and finally consolidate their skills through case discussions rooted in clinical practice [20]. Several experimental studies have demonstrated the advantages of this integrated approach. For example, a meta‐analysis of randomized controlled trials (RCT) in Chinese medical education showed that hybrid LBL‐PBL teaching significantly improved theoretical knowledge, clinical skills, and student satisfaction compared with traditional LBL alone [21]. Similarly, a RCT involving general practice trainees found that a CBL‐PBL combined model enhanced clinical thinking ability and led to higher knowledge test scores than conventional instruction [16]. Despite the theoretical appeal and emerging evidence, rigorous comparisons among LBL, PBL + CBL, and fully integrated LBL + PBL + CBL models remain limited—especially in the context of surgical subspecialty education. Most existing studies are observational or lack robust design, and few have been conducted in the Chinese context, where medical education is undergoing active reform to align with global standards [22].

To address this gap, we conducted a prospective, RCT to compare the effects of three different instructional models on gastrointestinal surgery trainees in a tertiary teaching hospital in China. The primary goal was to evaluate which teaching strategy most effectively enhances critical thinking, theoretical knowledge, learning efficiency, and comprehensive clinical competence.

## 2. Methods

### 2.1. Study Design and Participants

This prospective, RCT was conducted to compare the effects of three instructional models on the critical thinking ability, theoretical knowledge, learning efficiency, and clinical competence of gastrointestinal surgical trainees. The study was carried out at the Department of Gastrointestinal Surgery, Affiliated Hospital of North Sichuan Medical College between September 1 and November 1, 2024. The trial was registered in the Chinese Clinical Trial Registry (ChiCTR2000041569).

Inclusion criteria: Enrollment in the gastrointestinal surgery internship or residency program; completion of basic surgical coursework; willingness and ability to attend all teaching sessions for the full 8‐week intervention period; provision of written informed consent.

Exclusion criteria: Prior participation in a PBL or CBL curriculum in the past 12 months; absence exceeding 10% of scheduled classes; incomplete participation in pre‐ or post‐intervention assessments; withdrawal of consent at any time during the study.

### 2.2. Data Collection

Baseline demographic data (age, gender, educational background, prior academic performance [grade point average {GPA}], career interest, and learning motivation) were collected at enrollment using standardized forms. Pre‐ and post‐intervention assessments were conducted in the same week for all participants to ensure comparability. Written and oral examinations were graded by blinded assessors. All data were double‐entered and cross‐checked by two independent research assistants to ensure accuracy. Missing data were handled using listwise deletion during statistical analysis.

### 2.3. Randomization

A total of 120 trainees—including final‐year medical interns and first‐year surgical residents—participating in a gastrointestinal surgery rotation were enrolled in the study. After baseline data collection, participants were randomly assigned to one of three instructional groups in a 1:1:1 ratio (*n* = 40 per group): Group A: LBL; Group B: PBL + CBL; and Group C: integrated LBL + PBL + CBL. Randomization was performed using a simple computer‐generated sequence. Specifically, participants were first assigned a unique study ID based on enrollment order. A random number between 0 and 1 was then generated for each participant using Microsoft Excel′s. The list was sorted in ascending order based on the random number, and the first 40 participants were assigned to Group A, the next 40 to Group B, and the remaining 40 to Group C. The randomization procedure was performed by a research assistant who was not involved in data collection or instruction, to ensure allocation concealment. No stratification was used. After group assignment, the baseline characteristics of participants were compared across the three groups to confirm successful randomization and baseline equivalence.

### 2.4. Instructional Interventions

#### 2.4.1. LBL Group

The LBL group followed a conventional, teacher‐centered instructional model designed to deliver foundational knowledge in a structured and time‐efficient format. Each week, students attended three 2‐h classroom lectures (totaling 6 h/week) delivered by senior gastrointestinal surgery faculty. Lectures were thematically organized around core surgical topics and were delivered using PowerPoint slides supplemented with clinical images, procedural videos, and whiteboard drawings. Content focused on factual knowledge, including pathophysiology, operative techniques, perioperative protocols, and complication prevention. Students were required to take notes during class, engage in scheduled question and answer sessions, and complete assigned textbook readings and quizzes independently after each lecture. No group‐based learning or clinical case analysis was incorporated into this model. The instructor determined the depth and pacing of instruction, and students primarily assumed a passive role as recipients of information. Each week concluded with a summary of key concepts to support exam preparation.

#### 2.4.2. PBL + CBL Group

In the PBL component, each week began with a clinical scenario introduced to small groups of five–six students. These scenarios were deliberately open‐ended and clinically realistic, such as a patient presenting with signs of peritonitis or gastrointestinal bleeding. Students were responsible for identifying the key clinical questions, defining their own learning objectives, dividing research tasks, and consulting relevant literature, clinical guidelines, and textbook resources. Groups met twice weekly for 90‐min sessions in a dedicated discussion room. A trained faculty tutor facilitated the sessions, encouraged discussion, and ensured progress, but avoided providing direct answers or lecture‐style teaching. Each group presented their final diagnostic and management plan to peers and received formative feedback.

In the CBL component, students participated in structured case discussions later in the same week. Faculty provided real, deidentified clinical cases that included comprehensive information such as patient history, physical examination findings, laboratory and imaging results, intraoperative reports, and clinical outcomes. A five‐step worksheet was used to guide discussion: (1) identifying key problems, (2) interpreting clinical data, (3) forming differential diagnoses, (4) proposing management plans, and (5) evaluating outcomes and complications. CBL sessions were conducted twice weekly for 90 min each and were led by clinical instructors. Students were encouraged to justify their decisions, reflect on the clinical process, and discuss communication and ethical considerations. A written summary and group presentation were required at the end of each session. This instructional model emphasized inquiry, synthesis, and contextual application, without providing formal didactic input.

#### 2.4.3. Integrated LBL + PBL + CBL Group

The integrated group received a hybrid instructional design combining the structure of LBL with the active learning features of both PBL and CBL. Each week followed a three‐phase cycle aimed at sequentially guiding students from knowledge acquisition to application and clinical reasoning.

In Phase 1 (LBL), students attended a focused 120‐min lecture at the beginning of the week. These lectures were designed to provide foundational knowledge relevant to upcoming cases and problems. Faculty presented surgical concepts such as operative indications, anatomic landmarks, or complication management using concise slides, diagrams, and clinical examples. The lectures served as cognitive scaffolding to support subsequent problem‐solving and case analysis.

In Phase 2 (PBL), students worked in groups of five–six to explore a new clinical scenario without a predefined answer. They identified learning objectives based on the scenario, independently searched the literature, and collaborated to propose diagnostic and management strategies. Tutors facilitated the sessions by prompting deeper reasoning and ensuring balanced group participation, without directly supplying content. These sessions lasted approximately 120 min and emphasized analytical thinking and evidence‐based decision‐making.

In Phase 3 (CBL), students participated in structured case discussions that built upon their PBL work and integrated lecture content. Each CBL session lasted approximately 120 min. Using real clinical cases, students analyzed decision points, justified their reasoning, reflected on outcomes, and discussed alternative strategies. Faculty guided the discussion, clarified misconceptions, and encouraged students to consider the humanistic and ethical dimensions of care. Each group submitted a written summary and gave a brief presentation at the end of the week.

All participants received instruction over the same 8‐week gastrointestinal surgery curriculum, which covered major topics such as gastrointestinal anatomy, tumor staging, surgical indications, emergency procedures, perioperative management, and common postoperative complications. To ensure instructional consistency and reduce variability related to teaching style, all teaching sessions—including lectures, problem‐based discussions, and case‐based analyses—were delivered or facilitated by the same team of four senior faculty members from the gastrointestinal surgery department. Each faculty member underwent preparatory training and participated in the design of the teaching materials and case content to ensure unified instructional goals, timing, and difficulty level. For the integrated and PBL + CBL groups, faculty serving as discussion facilitators were trained in PBL/CBL methodology and followed standardized facilitation guidelines. Rotations among facilitators were minimized, and no faculty member crossed between intervention groups during the study period. Student attendance was strictly monitored throughout the 8‐week intervention. Participants were required to attend at least 90% of scheduled sessions.

### 2.5. Outcome Measures

The study evaluated four primary outcome domains: critical thinking ability, theoretical knowledge mastery, learning efficiency and learner satisfaction, and comprehensive clinical competence. Four outcome domains were systematically and comprehensively evaluated after the 8‐week instructional intervention. Validated instruments and standardized procedures were employed to ensure objective and reliable assessment across all groups.

Critical thinking ability was assessed using the Chinese version of the California critical thinking disposition inventory (CTDI‐CV) [23]. This 70‐item questionnaire evaluates seven key dimensions of critical thinking: truth‐seeking, open‐mindedness, analyticity, systematicity, inquisitiveness, self‐confidence, and maturity of judgment—each comprising 10 items. All items are scored on a 6‐point Likert scale ranging from 1 (*strongly disagree*) to 6 (*strongly agree*), yielding subscale scores that range from 10 to 60 and a total score ranging from 70 to 420. Higher scores indicate a stronger disposition toward critical thinking. According to the established grading standard, a total score of 280 or above was considered indicative of excellent critical thinking ability. In addition to the total score, individual subscale scores were analyzed. The CTDI‐CV is widely used in medical education research in China and has demonstrated robust psychometric properties, including high internal consistency and construct validity [24, 25].

Theoretical knowledge mastery was measured through a comprehensive summative examination designed by a panel of senior gastrointestinal surgeons [26]. This assessment comprised two components: a written examination and an oral examination. The written exam had a total score of 100 points and included two sections. The basic knowledge section (0–40 points) assessed core surgical concepts, principles, and terminology using multiple‐choice questions, matching items, and short‐answer questions. The case analysis section (0–60 points) required interpretation of clinical information (history, physical findings, laboratory, and imaging results) and application of surgical decision‐making skills in scenario‐based questions. Higher scores in each section and in the written exam total score indicated better mastery of theoretical knowledge. The oral exam (1–10 points) was conducted as a structured viva voce. Each student was presented with two real patient cases and was required to describe the diagnostic reasoning process, prioritize key clinical problems, and justify proposed management plans. Performance was evaluated using a standardized rubric covering accuracy of medical knowledge, logical reasoning, clinical relevance, and clarity of communication. All assessments were independently scored by two experienced faculty members who were blinded to the participants′ group assignments to reduce bias. When discrepancies occurred, scores were discussed and a consensus rating was reached.

Learning efficiency was evaluated using two indicators: total study time and task completion rate. Total study time was recorded as the average number of hours spent on gastrointestinal surgery learning activities per week during the 8‐week intervention, based on self‐maintained daily logs that were checked weekly by tutors to improve accuracy. The task completion rate was calculated as the percentage of assigned learning tasks, case reports, and reading assignments submitted on time each week, yielding a value between 0% and 100%. Higher percentages indicated better completion performance. Learner satisfaction was measured using a previously validated questionnaire [27] consisting of four items rated on a 5‐point Likert scale (1 = *very dissatisfied* to 5 = *very satisfied*), assessing clarity of teaching content, effectiveness of instruction, engagement during learning activities, and perceived improvement in clinical reasoning and competence. The total satisfaction score ranged from 1 to 5, with higher scores indicating greater learning efficiency and instructional satisfaction. Prior studies have shown that this instrument has acceptable internal consistency and content validity in higher‐education settings [28, 29].

Comprehensive clinical competence was measured using the miniclinical evaluation exercise (Mini‐CEX) [30]. Faculty members directly observed each student during routine clinical encounters and assessed performance across six domains: medical history taking, physical examination technique, clinical reasoning and decision‐making, demonstration of humanistic care and empathy, teamwork and collaboration, and overall clinical performance. Each domain was scored on a 9‐point scale, where 1–3 indicated *unsatisfactory performance*, 4–6 indicated *satisfactory performance*, and 7–9 indicated *excellent performance*. To ensure the consistency and reliability of assessment, all evaluating faculty received prior training in the use of the Mini‐CEX and remained blinded to group allocation throughout the study. Two experienced supervisors independently observed and scored each student, with discrepancies resolved by consensus. Prior studies have shown that this instrument demonstrates acceptable internal consistency and content validity in medical education settings in China, supporting its use for assessing clinical competence [31, 32].

### 2.6. Statistical Analysis

Statistical analysis was performed using IBM SPSS Statistics Version 26.0 (IBM Corp., Armonk, NY, United States). Continuous variables were presented as mean ± standard deviation (SD). Between‐group comparisons were conducted using one‐way analysis of variance (ANOVA). When significant differences were detected, Bonferroni post hoc tests were used for pairwise comparisons. Categorical variables were analyzed using the chi‐square test or Fisher′s exact test when expected cell counts were less than five. All tests were two‐tailed, and a *p* value of < 0.05 was considered statistically significant.

## 3. Results

### 3.1. Baseline Characteristics

**Figure 1:**
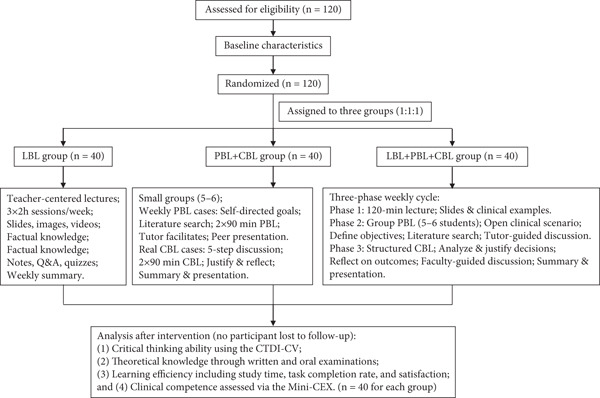
Flowchart of training process for three groups.

A total of 120 participants were enrolled in the study and successfully completed the 8‐week instructional intervention and all related assessments. No participant withdrew or was lost to follow‐up during the study period. A participant flow diagram is shown in Figure 1. Baseline demographic and academic characteristics were statistically comparable across the three groups, including age, gender, educational background, GPA, career interest, and self‐reported learning motivation (Table 1, all *p* > 0.05), indicating that randomization effectively established baseline equivalence and internal validity for group comparisons.

**Table 1:** Baseline characteristics of the study participants.

**Variables**	**LBL group (** **n** = 40**)**	**P** **B** **L** + **C** **B** **L** **group (** **n** = 40**)**	**L** **B** **L** + **P** **B** **L** + **C** **B** **L** **group (** **n** = 40**)**	**F**/**χ** ^2^	**p**
Age (years)	24.53 ± 1.24	24.33 ± 1.58	24.38 ± 1.44	0.213	0.809
Gender (male/female)	27/13	23/17	22/18	1.458	0.482
Education background (years)	17.40 ± 1.26	17.33 ± 1.31	17.40 ± 1.26	0.046	0.955
GPA score (0–4)	3.45 ± 0.23	3.47 ± 0.23	3.47 ± 0.21	0.169	0.845
Career interest score (1–10)	8.18 ± 1.03	8.30 ± 1.09	8.05 ± 1.26	0.487	0.616
Learning motivation score (1–10)	7.75 ± 1.37	8.08 ± 1.07	7.70 ± 1.24	1.086	0.341

Abbreviations: CBL, case‐based learning; GPA, grade point average; LBL, lecture‐based learning; PBL, problem‐based learning.

### 3.2. Critical Thinking Ability

As summarized in Table 2, significant differences in total critical thinking ability and its subdimensions were observed among the three groups, as determined by one‐way ANOVA (*p* < 0.001 for all comparisons). Specifically, the LBL + PBL + CBL group obtained the highest total score, followed by the PBL + CBL group, whereas the LBL group had the lowest score (*p* < 0.05). A similar trend was observed for analyticity, maturity of judgment, open‐mindedness, systematicity, inquisitiveness, and self‐confidence. In each of these domains, the LBL + PBL + CBL group achieved the highest mean values, the PBL + CBL group ranked second, and the LBL group had the lowest scores, and all between‐group comparisons were statistically significant (*p* < 0.05). For truth‐seeking, no significant difference was observed between the PBL + CBL and LBL groups (*p* > 0.05), whereas the LBL + PBL + CBL group still obtained significantly higher scores than both groups (*p* < 0.05).

**Table 2:** Comparison of critical thinking ability among the three groups.

**Outcomes**	**LBL group (** **n** = 40**)**	**P** **B** **L** + **C** **B** **L** **group (** **n** = 40**)**	**L** **B** **L** + **P** **B** **L** + **C** **B** **L** **group (** **n** = 40**)**	**F**	**p**
CTDI‐CV total score (70–420)	285.90 ± 10.08	317.65 ± 9.63^a^	359.05 ± 9.28^bc^	575.764	< 0.001
Analyticity score (10–60)	40.15 ± 4.52	44.38 ± 4.26^a^	49.63 ± 3.87^bc^	50.554	< 0.001
Maturity of judgment score (10–60)	40.05 ± 3.41	45.15 ± 3.13^a^	51.58 ± 2.38^bc^	147.705	< 0.001
Open‐mindedness score (10–60)	39.65 ± 3.31	44.08 ± 2.76^a^	49.08 ± 2.88^bc^	99.423	< 0.001
Truth‐seeking score (10–60)	41.55 ± 2.97	42.30 ± 2.71	47.40 ± 3.86^bc^	39.129	< 0.001
Systematicity score (10–60)	41.90 ± 3.18	47.08 ± 3.00^a^	52.80 ± 3.50^bc^	113.779	< 0.001
Inquisitiveness score (10–60)	43.18 ± 3.03	49.23 ± 3.22^a^	55.20 ± 3.25^bc^	143.969	< 0.001
Self‐confidence score (10–60)	39.43 ± 3.04	45.45 ± 3.37^a^	53.38 ± 2.80^bc^	206.567	< 0.001

*Note:* Data are presented as mean ± standard deviation (SD). *F*: one ‐way ANOVA test statistic. *p* value obtained from ANOVA; *p* < 0.05. indicates a statistically significant difference among the three groups. Post hoc comparisons were performed to determine between‐group differences.

Abbreviations: CBL, case‐based learning; LBL, lecture‐based learning; PBL, problem‐based learning; CTDI‐CV, Chinese version of the California critical thinking disposition inventory.

^a^The PBL + CBL group significantly differs from the LBL group.

^b^The LBL + PBL + CBL group significantly differs from the LBL group.

^c^The LBL + PBL + CBL group significantly differs from the PBL + CBL group.

### 3.3. Theoretical Knowledge Mastery

Table 3 presented the comparison of theoretical knowledge mastery among the three groups. One‐way ANOVA revealed statistically significant differences in all four assessment indicators, including the written exam total score, basic knowledge score, case analysis score, and oral exam score (*p* ≤ 0.001 for all). In terms of written exam total score, the LBL + PBL + CBL group scored significantly higher than both the PBL + CBL group and the LBL group (*p* < 0.05). However, no significant difference was observed between the LBL and PBL + CBL groups (*p* > 0.05). For basic knowledge, the LBL group had the highest score, and both the LBL + PBL + CBL and LBL groups scored significantly higher than the PBL + CBL group (*p* < 0.05). In case analysis, the PBL + CBL and LBL + PBL + CBL groups both outperformed the LBL group (*p* < 0.05). In the oral exam, the LBL + PBL + CBL group showed significantly higher scores than the LBL group (*p* < 0.05), whereas the difference between the LBL and PBL + CBL groups was not statistically significant (*p* > 0.05).

**Table 3:** Comparison of theoretical knowledge mastery among the three groups.

**Outcomes**	**LBL group (** **n** = 40**)**	**P** **B** **L** + **C** **B** **L** **group (** **n** = 40**)**	**L** **B** **L** + **P** **B** **L** + **C** **B** **L** **group (** **n** = 40**)**	**F**	**p**
Written exam total score (0–100)	87.88 ± 3.67	87.10 ± 3.72	90.03 ± 3.55^bc^	6.903	0.001
Basic knowledge score (0–40)	36.28 ± 2.08	33.45 ± 2.61^a^	35.15 ± 2.12^c^	15.545	< 0.001
Case analysis score (0–60)	51.60 ± 2.84	53.65 ± 2.88^a^	54.88 ± 2.72^b^	13.852	< 0.001
Oral exam score (1–10)	7.60 ± 1.06	7.98 ± 0.97	8.43 ± 0.90^b^	7.107	0.001

*Note:* Data are presented as mean ± standard deviation (SD). *F*: one‐way ANOVA test statistic. *p* value obtained from ANOVA; *p* < 0.05 indicates a statistically significant difference among the three groups. Post hoc comparisons were performed to determine between‐group differences.

Abbreviations: CBL, case‐based learning; LBL, lecture‐based learning; PBL, problem‐based learning.

^a^The PBL + CBL group significantly differs from the LBL group.

^b^The LBL + PBL + CBL group significantly differs from the LBL group.

^c^The LBL + PBL + CBL group significantly differs from the PBL + CBL group.

### 3.4. Learning Efficiency and Satisfaction

As shown in Table 4, significant differences were found among the three groups in total study time, task completion rate, and satisfaction score (all *p* < 0.001). The LBL group reported the longest weekly study time, whereas both the PBL + CBL and LBL + PBL + CBL groups spent significantly less time, with the integrated model showing the shortest study time (*p* < 0.05 for all pairwise comparisons). For task completion rate, the LBL + PBL + CBL group achieved the highest values, which were significantly higher than both the PBL + CBL and LBL groups (*p* < 0.05), whereas the difference between the latter two groups did not reach statistical significance (*p* > 0.05). Regarding student satisfaction, the LBL + PBL + CBL group reported the highest scores, followed by the PBL + CBL group, and the LBL group scored lowest, with all pairwise comparisons showing statistically significant differences (*p* < 0.05).

**Table 4:** Comparison of learning efficiency and satisfaction among the three groups.

**Outcomes**	**LBL group (** **n** = 40**)**	**P** **B** **L** + **C** **B** **L** **group (** **n** = 40**)**	**L** **B** **L** + **P** **B** **L** + **C** **B** **L** **group (** **n** = 40**)**	**F**	**p**
Total study time (h/week)	13.80 ± 2.41	11.60 ± 2.04^a^	10.18 ± 2.21^bc^	27.015	< 0.001
Task completion rate (%)	88.80 ± 4.31	91.10 ± 4.89	94.48 ± 4.18^bc^	16.310	< 0.001
Satisfaction score (0–5)	3.88 ± 0.61	4.25 ± 0.63^a^	4.63 ± 0.54^bc^	15.955	< 0.001

*Note:* Data are presented as mean ± standard deviation(SD). *F*: one‐way ANOVA test statistic. *p* value obtained from ANOVA; *p* < 0.05 indicates a statistically significant difference among the three groups. Post hoc comparisons were performed to determine between‐group differences.

Abbreviations: CBL, case‐based learning; LBL, lecture‐based learning; PBL, problem‐based learning.

^a^The PBL + CBL group significantly differs from the LBL group.

^b^The LBL + PBL + CBL group significantly differs from the LBL group.

^c^The LBL + PBL + CBL group significantly differs from the PBL + CBL group.

### 3.5. Comprehensive Clinical Competence

Table 5 compared the comprehensive ability scores of students across the three instructional groups. One‐way ANOVA revealed significant differences among the groups for all six Mini‐CEX indicators (*p* < 0.001 for all). The LBL + PBL + CBL group achieved the highest overall clinical performance score, followed by the PBL + CBL group, whereas the LBL group had the lowest score, and all pairwise comparisons were statistically significant (*p* < 0.05). A similar graded pattern was found in history taking, clinical reasoning, humanistic care, and team collaboration, where the LBL + PBL + CBL group scored highest, the PBL + CBL group ranked second, and the LBL group showed the lowest performance, with all between‐group differences reaching statistical significance (*p* < 0.05). For physical examination, the integrated group scored significantly higher than both the PBL + CBL and LBL groups (*p* < 0.05), whereas the difference between the latter two groups was not significant (*p* > 0.05).

**Table 5:** Comparison of comprehensive ability among the three groups.

**Outcomes**	**LBL group (** **n** = 40**)**	**P** **B** **L** + **C** **B** **L** **group (** **n** = 40**)**	**L** **B** **L** + **P** **B** **L**+ **group (** **n** = 40**)**	**F**	**p**
Overall clinical performance (1–9)	6.28 ± 0.45	6.95 ± 0.32^a^	7.88 ± 0.40^bc^	105.156	< 0.001
History taking score (1–9)	5.93 ± 0.73	6.60 ± 0.74^a^	7.50 ± 0.64^bc^	50.057	< 0.001
Physical examination score (1–9)	6.65 ± 0.74	6.93 ± 0.62	8.00 ± 0.60^bc^	47.758	< 0.001
Clinical reasoning score (1–9)	6.18 ± 0.64	7.03 ± 0.58^a^	7.98 ± 0.77^bc^	73.363	< 0.001
Humanistic care score (1–9)	6.75 ± 0.74	6.98 ± 0.70	7.63 ± 0.70^bc^	16.144	< 0.001
Team collaboration score (1–9)	6.70 ± 0.65	7.08 ± 0.62^a^	8.25 ± 0.90^bc^	48.832	< 0.001

*Note:* Data are presented as mean ± standard deviation (SD). *F*: One‐way ANOVA test statistic. *p* value obtained from ANOVA; *p* < 0.05 indicates a statistically significant difference among the three groups. Post hoc comparisons were performed to determine between‐group differences.

Abbreviations: CBL, case‐based learning; LBL, lecture‐based learning; PBL, problem‐based learning; Mini‐CEX, miniclinical evaluation exercise.

^a^The PBL + CBL group significantly differs from the LBL group.

^b^The LBL + PBL + CBL group significantly differs from the LBL group.

^c^The LBL + PBL + CBL group significantly differs from the PBL + CBL group.

## 4. Discussion

Although various instructional approaches are used in surgical education, there is still limited evidence on whether a unified model that combines LBL, PBL, and CBL offers added benefits in gastrointestinal surgery [26]. This study demonstrated that an integrated instructional model combining LBL, PBL, and CBL significantly enhanced gastrointestinal surgical trainees′ critical thinking ability, theoretical knowledge mastery, learning efficiency and satisfaction, and clinical competence compared with either LBL alone or PBL + CBL.

This study demonstrated that the integrated LBL + PBL + CBL model most effectively enhanced critical thinking among gastrointestinal surgery trainees, as evidenced by significantly higher CTDI‐CV scores across all subscales. The initial lecture component helped reduce extraneous cognitive load by establishing conceptual foundations, enabling students to engage more effectively in the reasoning and synthesis required during PBL and CBL sessions [21]. During PBL, learners were required to generate hypotheses, evaluate evidence, and justify their reasoning, whereas CBL provided clinical context through authentic scenarios that demanded decision‐making under uncertainty [16, 33]. This sequential design is consistent with cognitive load theory and constructivist learning principles, which emphasize the role of structured scaffolding in supporting higher‐order reasoning [34]. Consistent with prior comparative studies showing that problem‐oriented teaching approaches enhance critical thinking more effectively than traditional didactic instruction [15, 35], the present study further demonstrates that a systematically integrated and scaffolded instructional model can be effectively applied in the context of gastrointestinal surgical training.

The integrated group also achieved the highest overall theoretical knowledge scores, particularly in case analysis and oral examinations. Although basic knowledge scores were highest in the LBL group, suggesting that lecture‐based instruction remains more effective for delivering structured, factual content [6]. PBL and CBL encourage integration of concepts and provide opportunities for cognitive rehearsal in realistic contexts, promoting knowledge transfer [18, 36]. These findings are consistent with research showing that although didactic instruction promotes declarative knowledge, applied learning is necessary for procedural and clinical reasoning [37]. Thus, foundational lectures and active learning are not competing strategies but complementary phases of a unified process [18]. The hybrid approach thus offers a pedagogically balanced solution—leveraging lectures to anchor core concepts and active learning to promote flexible application in complex clinical contexts [21].

Despite reporting the shortest average study time per week, students in this group achieved the highest task completion rate and satisfaction score. From the perspective of self‐determination theory, this may reflect fulfillment of learners′ psychological needs [38]. In the integrated model, competence was supported through clear conceptual frameworks provided by LBL, autonomy was fostered through hypothesis‐driven inquiry in PBL, and relatedness was strengthened through the collaborative interactions embedded in CBL [39, 40]. Prior research has shown that when these needs are met, learners demonstrate higher intrinsic motivation, deeper engagement, and more meaningful learning behaviors, which may explain both the higher task completion rates and the elevated satisfaction reported in this study [41]. This efficiency is particularly relevant in clinical education settings, where time constraints and workload demand instructional models that optimize both outcomes and effort.

In clinical performance, the integrated group outperformed both comparison groups across all Mini‐CEX domains, demonstrating superior transfer of knowledge to practice. Improvements in history taking and physical examination may stem from the sequential buildup of conceptual knowledge through LBL, followed by hypothesis‐driven inquiry in PBL, which encourages learners to organize clinical cues systematically before applying them in authentic case scenarios [42]. The enhancement in clinical reasoning likely reflects the cumulative effect of guided inquiry and case interpretation, during which learners must synthesize disparate pieces of information, weigh differential diagnoses, and justify decisions [43]. The CBL component played a central role in this process by situating learning within realistic patient narratives that require communication, perspective‐taking, and collaborative problem solving [44]. When combined with the preparatory scaffolding offered by LBL and PBL, these case‐based activities formed a logically sequenced learning pathway, consistent with evidence that blended instructional models enhance applied clinical competence when their components are intentionally structured [45, 46]. Moreover, the model′s ability to support both cognitive and interpersonal aspects of practice aligns with previous findings that blended approaches promote more effective performance in patient‐centered clinical environments [47].

Our findings are consistent with and extend those of previous studies that have explored the benefits of active and blended learning in medical education. For instance, Oja [48] has emphasized that PBL improves students′ ability to integrate knowledge and apply it to clinical problems. Similarly, Safa et al. [15] have demonstrated that structured PBL approaches were more effective than traditional LBL method in improving nursing students′ critical thinking. Sartania et al. [49] have concluded that CBL promotes learner engagement and clinical reasoning but may vary in effectiveness depending on case design and facilitation. These insights align with our results, which shows that PBL + CBL improves outcomes but is less effective than when preceded by targeted didactic instruction. Hafizah et al. [50] have conducted a meta‐analysis showing that PBL contributes positively to physicians′ critical thinking and clinical decision‐making, particularly when supported by structured content. Our study confirms and builds on this by showing that when LBL is used to establish foundational understanding, the effectiveness of subsequent PBL and CBL activities is amplified. In a Chinese context, Yang et al. [51] have found that a flipped classroom combining short lectures and clinical cases improved critical thinking, further supporting the use of hybrid models. Notably, Jiang et al. [52] have reported that combining PBL and CBL improved clinical thinking among assistant general practitioners. However, their study did not include an LBL component. Our results suggest that the inclusion of LBL serves as a vital cognitive scaffold, allowing learners to better extract meaning from PBL and CBL activities. Together, these studies reinforce the conclusion that a balanced combination of teacher‐led and student‐centered approaches may yield optimal educational outcomes.

However, several limitations should be acknowledged. First, the study was conducted over an 8‐week period, and long‐term retention of learning outcomes was not assessed. Future studies should include follow‐up assessments during clinical rotations or early postgraduate training to evaluate sustained effects. Second, although the sample size was adequate for primary comparisons, subgroup analyses (e.g., by learning style, baseline motivation, or prior academic ability) were not performed. Exploring such interactions could provide more personalized guidance for instructional design. Third, this study relied primarily on quantitative outcome measures. Incorporating qualitative data—such as learner reflections, focus group interviews, or faculty observations—could yield deeper insights into learner experiences and perceptions of value. Additionally, this study focused exclusively on gastrointestinal surgery, replicating the study across other specialties would help validate the model′s generalizability.

## 5. Conclusion

This RCT demonstrated that an integrated LBL + PBL + CBL instructional model significantly improves critical thinking, theoretical knowledge, learning efficiency and satisfaction, and clinical competence among gastrointestinal surgery trainees. Compared with LBL alone or PBL + CBL, the hybrid model achieved superior outcomes across all domains. Structured lectures provided foundational knowledge that enhanced the effectiveness of problem‐ and case‐based learning. These findings support the adoption of integrated teaching strategies in surgical education to foster both cognitive development and practical skills. Future studies should explore long‐term retention and model applicability across specialties and educational settings.

**Nomenclature**
LBL: lecture‐based learning
PBL: problem‐based learning
CBL: case‐based learning
CTDI‐CV: California critical thinking disposition inventory
Mini‐CEX: miniclinical evaluation exercise
RCT: randomized controlled trials
GPA: grade point average
SD: standard deviation
ANOVA: analysis of variance

## Ethics Statement

The study was approved by the ethics committee of the Affiliated Hospital of North Sichuan Medical College (Approval Number: 2020ER (N) 175‐1). Written informed consent was obtained from all individuals included in this study. All procedures were conducted in accordance with the Declaration of Helsinki.

## Disclosure

All authors read and approved the final manuscript.

## Conflicts of Interest

The authors declare no conflicts of interest.

## Author Contributions

Jin Tang is responsible for the guarantor of integrity of the entire study, study concepts and design, definition of intellectual content, clinical studies, experimental studies, data acquisition, statistical analysis, and manuscript editing; Jing Yang is responsible for the guarantor of integrity of the entire study, study concepts, study design, definition of intellectual content, clinical studies, experimental studies, data acquisition and analysis, and statistical analysis; Junsong Yang is responsible for the study concepts and design, literature research, experimental studies, data acquisition, and statistical analysis; Shoujiang Wei is responsible for the study concepts and manuscript review. Jin Tang and Jing Yang are the co‐first authors and contributed equally to this work.

## Funding

This study was supported by the Sichuan Provincial Science and Technology Department Key R&D Program (Major Science and Technology Special Project) (No. 2022YFS0168) and Special Scientific Research Project of Sichuan Medical Association (No. 2024HR08).

## Data Availability

The experimental data used to support the findings of this study are available from the corresponding author upon request.
